# Variant curation and interpretation in hereditary cancer genes: An institutional experience in Latin America

**DOI:** 10.1002/mgg3.2141

**Published:** 2023-03-10

**Authors:** María Carolina Manotas, Ana Lucia Rivera, María Carolina Sanabria‐Salas

**Affiliations:** ^1^ Medical Subdirection Instituto Nacional de Cancerología Bogotá Colombia; ^2^ Subdirection of Research Instituto Nacional de Cancerología Bogotá Colombia

**Keywords:** automated curation, hereditary cancer, manual curation, variant interpretation

## Abstract

**Background:**

Variant curation refers to the application of evidence‐based methods for the interpretation of genetic variants. Significant variability in this process among laboratories affects clinical practice. For admixed Hispanic/Latino populations, underrepresented in genomic databases, the interpretation of genetic variants for cancer risk is challenging.

**Methods:**

We retrospectively evaluated 601 sequence variants detected in patients participating in the largest Institutional Hereditary Cancer Program in Colombia. VarSome and PathoMAN were used for automated curation, and ACMG/AMP and Sherloc criteria were applied for manual curation.

**Results:**

Regarding the automated curation, 11% of the variants (64/601) were reclassified, 59% (354/601) had no changes in its interpretation, and the other 30% (183/601) presented conflicting interpretations. With respect to manual curation, of the 183 variants with conflicting interpretations, 17% (*N* = 31) were reclassified, 66% (*N* = 120) had no changes in their initial interpretation, and 17% (*N* = 32) remained with conflicting interpretation status. Overall, 91% of the VUS were downgraded and 9% were upgraded.

**Conclusions:**

Most VUS were reclassified as benign/likely benign. Since false‐positive and ‐negative results can be obtained with automated tools, manual curation should also be used as a complement. Our results contribute to improving cancer risk assessment and management for a broad range of hereditary cancer syndromes in Hispanic/Latino populations.

## Graphical abstract

An automated and manual curation workflow allows us to reclassify 16% (95/601) of the genetic variants identified in a Latin American population with a suspected hereditary cancer syndrome. VUS corresponded to 71% of the reclassified variants; from these, 91% were downgraded to B/LB and only 9% were upgraded to P/LP. Our results contribute to improving cancer risk assessment and management for a broad range of hereditary cancer syndromes in Hispanic/Latino populations.

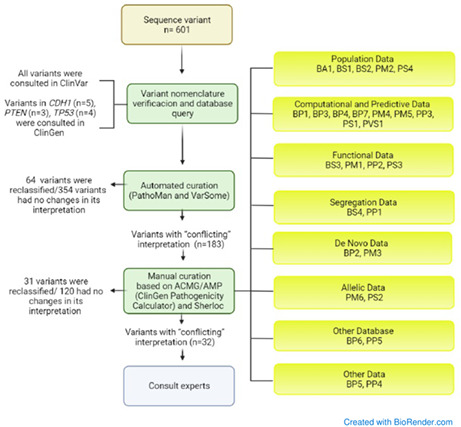

## 1 INTRODUCTION

Cancer genetics is becoming increasingly integrated into the practice of modern medical oncology (Guan et al., 2012; Kamps et al., 2017). Rapid advances in DNA‐sequencing technologies have brought great challenges to the scientific and medical community regarding the clinical use of genomic data to support precision medicine initiatives and improve medical care and management. It is noteworthy the great impact of massive parallel sequencing (such as next generation sequencing, NGS) in understanding the biological processes in oncological diseases and in personalizing care for this group of patients (Kamps et al., 2017). This includes its implementation in the study of hereditary cancer syndromes since these represent 5–10% of all cancer patients and the identification of these carriers is very important to give specific recommendations in the surveillance and prevention of malignant tumors (National Comprehensive Cancer Network, 2021; National Comprehensive Cancer Network, 2023; Owens et al., 2019).

Genetic testing has been used for patients with suspected hereditary cancer since the last decades in the United States and Europe (Garber & Offit, 2005; Guan et al., 2012) and increasingly in Latin America (Alvarez‐Gomez et al., 2021). Sanger sequencing is considered the “gold standard” method for DNA‐sequencing analysis and it was widely used for many years to determine an entire sequence and identify‐unknown genetic variants. Even today, it is used for validating positive genetic results identified by massive parallel sequencing (i.e., confirmation of specific pathogenic or likely pathogenic variants) (de Cario et al., 2020; Guan et al., 2012). However, this traditional method for genetic testing is expensive, time consuming, and low throughput (Guan et al., 2012; Walsh et al., 2010). On the other hand, the development of massive parallel sequencing has provided many opportunities to simultaneously detect in multiple genes several numbers and types of variants, such as single nucleotide substitutions (SNVs) and small insertions and deletions (INDELs); it is even possible to infer copy number variants (CNVs) from genetic sequencing data for the identification of large genomic duplications and deletions (Parilla & Ritterhouse, 2020).

The large amount of genetic data obtained with the implementation of parallel sequencing methods demand computational and bioinformatics skills to establish workflows aimed at improving the accuracy and interpretation of the identified genetic variants in a clinical setting (Pereira et al., 2020). Specifically, for the study of hereditary cancer syndromes, the clinical interpretation of DNA variants aims to assess the deleterious effect of a variant on gene function (i.e., pathogenicity of the variant), which is an important fact to explain its implications and give specific recommendations to the patient and relatives in the context of genomic risk assessment and counseling (Zhang et al., 2020). In 2015, the American College of Medical Genetics and Genomics (ACMG) and the Association for Molecular Pathology (AMP) published standards and guidelines for the evaluation of the evidence in the clinical interpretation of sequence variants with increased consistency and transparency, and avoiding false associations with disease; the defined framework of five tiers for variants classification was as follows: 5—pathogenic (P), 4—likely pathogenic (LP), 3—variant of uncertain clinical significance (VUS), 2—likely benign (LP), or 1—benign (B) (Richards et al., 2015). Variants classified as P and LP are treated as clinically actionable and influence clinical recommendations and care, while variants in categories B, LB, and VUS are treated as non‐clinically actionable (Richards et al., 2015; Slavin et al., 2019). In 2017, the Invitae Clinical Genomics Group published a logical framework known as Sherloc that provides hierarchical evidence‐based rules for locus interpretation to achieve a consistent and reproducible classification of genes and variants based on the ACMG–AMP criteria with the introduction of over 100 detailed refinements (Nykamp et al., 2017).

ACMG/AMP guidelines provide a framework for evaluating the pathogenicity of variants, and clinical laboratories in different parts of the world have widely adopted these guidelines to lead their clinical interpretation (Zhang et al., 2020). Even though the guideline was developed to be broadly applicable across many genes, inheritance patterns, and diseases, it is encouraged “that those working in specific disease groups should continue to develop more focused guidance regarding the classification of variants in specific genes given that the applicability and weight assigned to certain criteria may vary by gene and disease” (Richards et al., 2015). As a result, an increasing number of disease‐specific variant expert panels, such as The ClinGen Sequence Variant Interpretation Working Group and several others, have been established with the purpose of generating disease‐/gene‐specific guidelines (Brnich et al., 2020; Ellard et al., 2020; Fortuno et al., 2021; Lee et al., 2018; Luo et al., 2019; Mester et al., 2018; Zhang et al., 2020).

Variant curation is a process that consists of the application of evidence‐based methods for the interpretation of sequence variants in a given gene. This process can be done manually or automated; while manual curation is the standard method, it can become time consuming on a large scale, necessitating automation that enables rapid processing with reproducible results (Pandey et al., 2012). Among the automated computational frameworks available for curation of germline genetic variants in cancer susceptibility genes, PathoMAN and VarSome stand out, as their algorithms are inspired by the ACMG/AMP classification and aggregate multiple genetic and molecular evidence using variant annotators and public repositories that contain data for the pathogenicity assertion (Kopanos et al., 2019; Ravichandran et al., 2019). PathoMAN classifies the collected data into 28 distinct categories that are grouped as: variant type, biological impact, in silico predictions, presence or frequency in controls, familial information, and mode of inheritance; the resulting score from the evaluation of these categories is used to generate the classification of the variant. The performance of PathoMAN was measured by retesting expert‐curated germline cancer variants from three clinical testing laboratories: Ambry, Invitae, and GeneDx, achieving a high overall concordance of 94.4% for pathogenic variants and 81.1% for benign variants (Ravichandran et al., 2019). On the other hand, VarSome includes information from external databases such as Clinical Interpretations of Variants in Cancer (CIVIC), International Agency for Research on Cancer TP53 (IARC TP53 Database), and International Cancer Genome Consortium (ICGC), among other 27 external databases. Overall, VarSome holds more than 33 billion data points describing more than 500 million variants, and to deal with this scale of data, they developed an extremely efficient data warehouse that maps a variant to a specific genomic location and identifies equivalent variants, the variant type (i.e., frameshift, insertion, deletion, etc.), and its effect (Kopanos et al., 2019).

Despite efforts to standardize the interpretation of variants, in clinical practice, there is still significant variability in the process of variant curation. We present the results of the retrospective reassessment of 601 gene sequence variants that were identified in cancer patients through the implementation of a Hereditary Cancer Program's Registry at the Instituto Nacional de Cancerología, Bogotá, Colombia (INC‐C), applying a workflow that integrates automated and manual curation with the aim of improving the accurate interpretation of sequence variants and updating their classification to institutional cancer patients.

## 2 METHODS

### 2.1 Identification of genetic variants in patients included in the Institutional Hereditary Cancer Program

The Scientific Committee of the INC‐C approved in 2017 the implementation of a Hereditary Cancer Program as a Quality Improvement Project to offer germline genetic testing as a standard‐of‐care service for cancer patients with suspicion of an underlying inherited condition. Patients who meet the criteria for a hereditary cancer syndrome according to the NCCN Guidelines or another international guideline if applicable (i.e., International Gastric Cancer Linkage Consortium, IGCLC) were admitted to the program. All participants in the institutional program accepted to be donors at the tissue Biobank from the INC‐C named “Banco Nacional de Tumores Terry Fox” and signed informed consent for DNA biobanking and future research studies. The Program's registry is being implemented for epidemiological reports and this variant curation exercise includes the genetic results collected from April 2018 to June 2020. Automated and/or manual curation was performed from December 01, 2020, to February 01, 2021, for 601 unique sequence variants identified through massive parallel sequencing and initially classified as follows: 100 P, 26 LP, 18 P/LP, and 457 VUS.

#### 2.1.1 DNA extraction, library preparation, and massive parallel sequencing (NGS)

Germline DNA was extracted from peripheral blood using the Quick‐DNA TM Miniprep Plus Kit (Zymo Research, USA) following the manufacturer's instructions. DNA was quantified with the Qubit dsDNA BR kit (Invitrogen) and the NanoDrop™ 2000 (Thermo Scientific) equipment, and DNA quality was assessed with the Bioanalyzer High Sensitivity DNA Analysis Kit (Agilent). Library preparation and sequencing were performed as recommended by the manufacturer's protocol for the Nextera Flex for Enrichment Illumina kit and the Canadian Consortia Inherited Cancer, a customized probe panel (reference # 20011891; Illumina Inc., San Diego, USA), that targets 105 genes known to be strongly associated with inherited cancers—or candidate genes—and detects single nucleotide variants (SNVs) and small insertions and deletions (INDELs) (Table S1). Additionally, the multigene panel design allows inferring possible copy number variants (CNVs) in all genes with bioinformatics methods by using sequencing data. Briefly, the DNA sample was enriched for the target regions using the protocol by Nextera Flex for Enrichment (Illumina Inc., San Diego, CA) based on hybridization, and the libraries were sequenced using paired‐end technology (2 × 251 cycles) in a MiSeq instrument (Illumina Inc., San Diego, CA). Unless otherwise indicated, all target regions were sequenced to a depth greater than 100X (if an allelic depth greater than 50X was not achieved, complementary analyses of the region of interest were performed by orthogonal methods). This assay focuses on the coding sequences of the genes included. Promoter regions, non‐transcribed regions, and other non‐coding regions are not included. Massive parallel sequencing was previously standardized and validated in our laboratories to analyze 12 samples simultaneously and, according to the Analytical Performance results, we obtained a sensitivity of 99.89% and a specificity of 99.99% for the accurate detection of SNVs and small INDELs genetic variants using the commercial bioinformatics software developed by Sophia Genetics (Saint‐Sulpice, Switzerland). CNV detection by massive parallel sequencing with the Sophia software has not been validated, so these variants are always confirmed at an external laboratory with Multiplex Ligation‐dependent Probe Amplification (MLPA) assays as this is the “Gold Standard” method to identify large genomic deletions and duplications.

#### 2.1.2 Variant calling and initial interpretation

Sequence reads in FastQC files were aligned to the hg19 human reference genome with the Burrows–Wheeler Aligner (BWA) tool. Variant calling and annotation of SNVs and INDELs were carried out with the SOPHiA DDM® platform using the ILL1IC1G3_TSC algorithm (Sophia Genetics, Saint‐Sulpice, Switzerland). This algorithm also allows inferring CNVs from sequence data for all genes included in the panel, and the detection of ALU elements. The genetic variants detected by the SOPHiA algorithm were reviewed by an oncogeneticist and a biologist trained in genetics for interpretation and delivery of genetic results at the post‐test consultation.

The Human Genome Variation Society (HGVS) nomenclature (http://www.hgvs.org/) was used in the genetic report, and the five‐tier criteria of the American College of Medical Genetics and Genomics (ACMG) for variant classification were implemented for variant classification (Richards et al., 2015).

### 2.2 Definition of the institutional workflow for the curation of *n* = 601 unique genetic variants identified in patients included in the Institutional Hereditary Cancer Program

**FIGURE 1:**
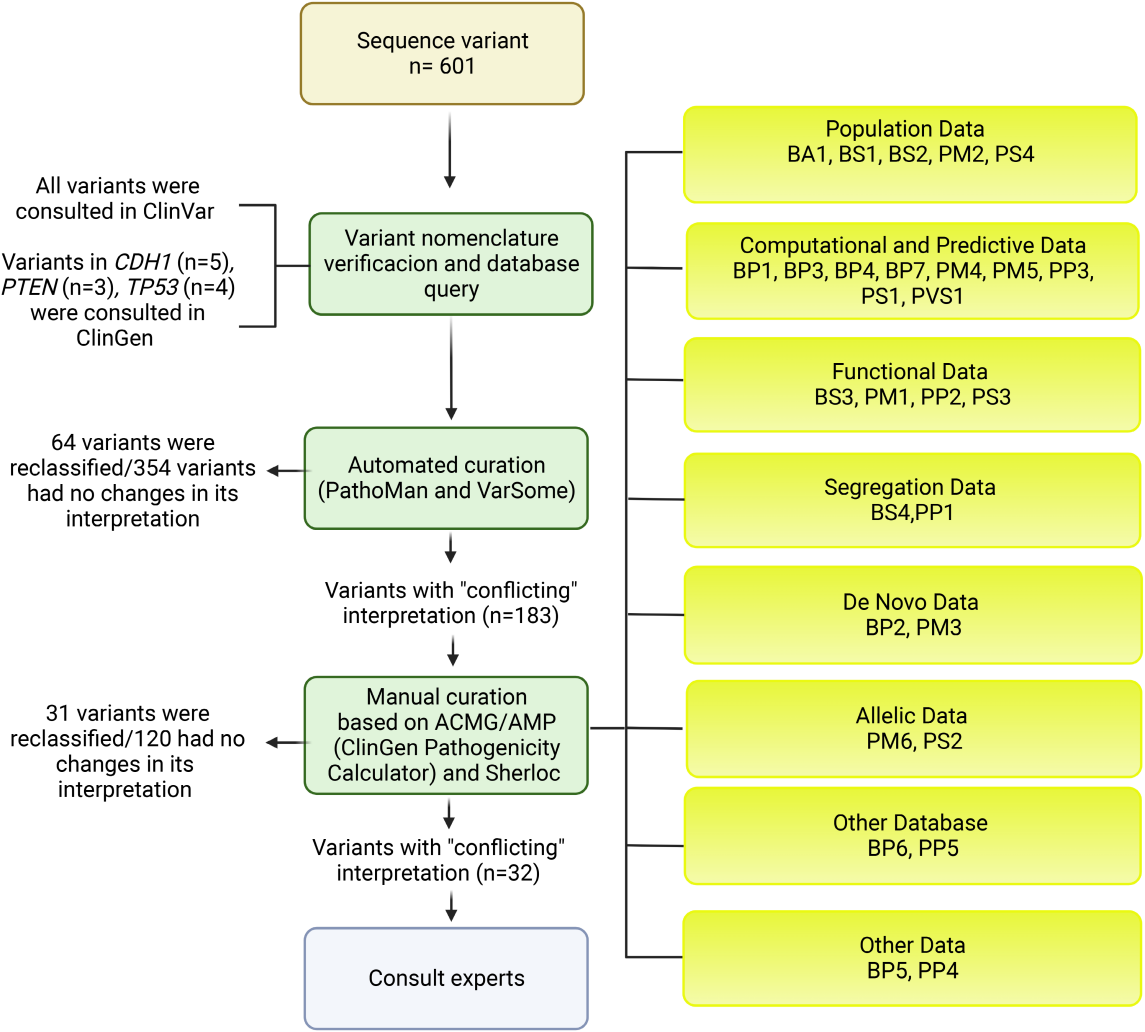
Workflow for interpretation of sequence variants. Overview of the variant curation process defined at the INC‐C to reclassify a total of 601 unique variants (light brown box). The workflow is divided into three sections: checks of sequence variant nomenclature and database interrogation; automated curation; and manual curation (light green boxes). Some variants with unresolved conflicting status after previous steps were further consulted with expert laboratories (i.e., Myriad and Invitae) (light blue box). ACMG/AMP criteria are summarized in bright yellow boxes.

A review of the literature as of December 01, 2020, was carried out followed by a discussion among the authors about the different tools available for genetic variant curation to define the institutional workflow to reassess and update the classification of the sequence variants listed in the Program's registry. The proposed workflow was divided into three sections: (B1) checks of sequence variant nomenclature and database interrogation; (B2) automated curation; and (B3) manual curation. Additionally, some selected genetic variants with unresolved conflicting interpretations with the previous steps were consulted with expert laboratories. Not for all the variants it was necessary to apply the three steps of the workflow. The interpretation was updated if the evidence was sufficient to reclassify the variant (Figure 1).

#### 2.2.1 Nomenclature verification and database interrogation

The variant nomenclature was verified according to criteria developed by the Human Genome Variation Society (den Dunnen et al., 2016). All the variants were consulted in the ClinVar database, considering the specific transcript of each gene. Variants in the *PTEN*, *CDH1*, or *TP53* genes were additionally consulted in the ClinGen repository. Reported interpretations were registered in a database designed in Excel.

#### 2.2.2 Automated curation

For automated curation, the platforms VarSome (free version) and PathoMAN were used. The 601 variants were queried on both platforms using the GRCh37 reference genome. Given that VarSome recognizes variants in many different formats, the search was performed using the nomenclature based on coding DNA (c.) reference sequences, always verifying the transcript and the effect on the protein. For PathoMAN, the information required for each variant query was filled out (chromosome, position, reference allele, alternative allele, allele count, allele number, de novo status, co‐segregation status, and preferred control population sub‐group). The allele counts and allele numbers were consulted in the Genome Aggregation Database (gnomAD) (Koch, 2020), using the option “All” as the control population. The results of the variants interpretation and the ACMG/AMP criteria used for each platform were recorded in an Excel database.

The pathogenicity criteria scored in VarSome and PathoMAN, as a result of the automated curation, were reviewed and compared to expert curator reports. Final results were reported in three categories: (i) variants without changes in interpretation; (ii) reclassified variants; and (iii) variants with conflicting interpretation. Concordance between the results of VarSome and PathoMAN was determined, and between these two with what was previously reported in our database according to our initial classification. When the classification of the variants was concordant in VarSome and PathoMAN with ours, the results were considered as variants without changes in interpretation. When VarSome and PathoMAN agreed on the reclassification of the variants as P, LP, VUS, LB, or B but differed from those reported in our database, the variants were reclassified. On the other hand, if the resulting reclassification of the variants were different in VarSome, PathoMAN, and our initial classification, the variant was considered to have a conflict of interpretation. Prior to concluding about the results in any of the three categories with the automated tools, manual input was required. Briefly, each one of the criteria indicated by VarSome and PathoMAN was verified with the help of various tools available online (i.e., genetic databases and predictors in silico) and the assessments performed by expert curators (if available) to confirm the evaluation obtained by each of these tools and resolve if a reclassification occurred or not.

#### 2.2.3 Manual curation

Variants with conflicting interpretations after the automated curation were curated manually. Manual curation was based on the application of the ACMG/AMP and Sherloc criteria. The ACMG/AMP guide establishes 16 criteria that help classify pathogenicity and 12 criteria that help classify benignity; this classification system is based on eight central components to resolve a variant as pathogenic or benign, which are population frequency data, genomic annotation and computational predictive data, functional data, segregation data, de novo data, allelic/genotypic data, public databases and literature, and other data (Ravichandran et al., 2019). The classification of the variants in one of the five tiers (i.e., P, LP, VUS, LB, or B), is based on the score built on the criteria that evaluate the levels of evidence of pathogenicity; each criterion has a code consisting of two or three letters and a number. The first letter indicates a direction, pathogenic (P) or benign (B); the following letter(s) denotes the level of strength: very strong (VS), strong (S), moderate (M), supportive (P), and independent (A); and the number acts as a serial digit to distinguish criteria of the same strength. Combinations of different pieces of evidence are used to determine the classification of a sequence variant (Richards et al., 2015). The ClinGen Pathogenicity Calculator was used for this manual curation based on the ACMG/AMP criteria, since it allows eliminating errors in the application of rules and allows automatic calculation of interim conclusions based on the latest evidence, with supporting web links (Patel et al., 2017). For the manual curation with Sherloc, the rules described in the original publication (i.e., 108 refinements of the ACMG/AMP criteria) were applied and an Excel file was generated for each variant with its respective score and classification (Nykamp et al., 2017).

Similarly, the results of the manual curation based on the ACMG/AMP and Sherloc criteria were reported in three categories: (i) variants without changes in interpretation, (ii) reclassified variants, and (iii) variants with conflicting interpretation. When the classification with the ACMG/AMP and Sherloc criteria matched with the one reported in our database, they were considered variants without changes in interpretation. If the returned classifications of the variants match using the ACMG/AMP and Sherloc criteria but differ from those reported in our database, they were considered reclassified variants. Finally, the interpretation was considered conflicting, if the results of the ACMG/AMP and Sherloc curation‐based variant classification did not agree with each other and/or with our initial classification.

Sequence variants were associated with phenotypes described in OMIM (Online Mendelian Inheritance in Man) and gnomAD was accessed to search for allelic frequencies (Koch, 2020). Many in silico predictor tools were used to assess the pathogenicity of exonic variants, including PolyPhen, SIFT, PROVEAN, Mutation Taster, and AlingGVGD. For intronic variants, the Ensembl Variant Effect Predictor (VEP) was used and the NCBI repository was consulted to obtain the protein sequence in FASTA format and its isoforms. *BRCA1* and *BRCA2* genetic variants were consulted in the FLOSSIES database which consists of a registry of germline variants from 10,000 women older than 70 years, who have never had cancer (including European Americans and African Americans). Also, the BRCA Exchange database was used to assess the PM5 and PS1 criteria in these genes. Regarding *CDH1* gene variants, the interpretation was made based on the recommendations published by a panel of experts (i.e., Variant Curation Expert Panel—VCEP) (Lee et al., 2018).

The search for complementary scientific literature was performed in PubMed and ScienceDirect, using the following generic terms: ‐name of the gene‐; ‐variant nomenclature in coding DNA‐; and ‐alternative variant nomenclatures accepted for the amino acid change‐.

### 2.3 Descriptive analyses and graphical software

Results of the genetic variant reclassification obtained with each tool were annotated in Excel datasheets and summarized using descriptive statistics. All the descriptive analyses and graphics were performed with R statistics v.4.1.3.

## 3 RESULTS

**FIGURE 2:**
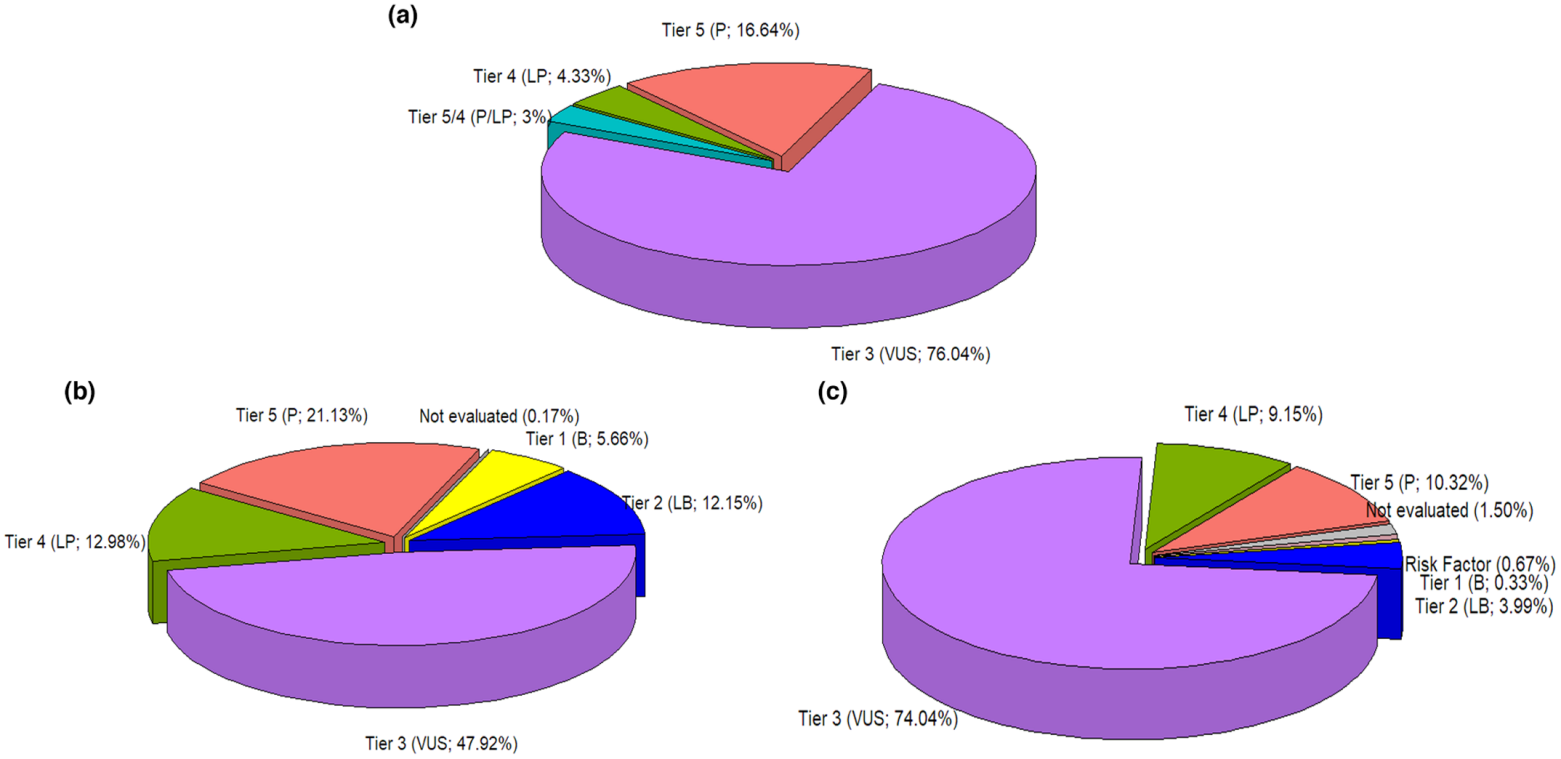
Overall distribution of genetic variant's classification by pathogenicity. (a) Initial classification at the INC‐C and results using (b) VarSome and (c) PathoMAN.

A total of 601 unique sequence variants in 92/105 genes (Figure S1) were identified in 607 patients of the Institutional Hereditary Cancer Program and were re‐evaluated according to the curation protocol defined here. Some patients had many VUS or a combination of one P/LP variant with one or more VUS or even two P/LP variants (data not shown). Most genetic variants were reported in the following genes: *BRCA2* (*n* = 34), *ATM* (*n* = 33), *RECQL4* (*n* = 26), *MSH6* (*n* = 25), *BRCA1* (*n* = 23), *FANCM* (*n* = 21), *CHEK2* (*n* = 20), *APC* (*n* = 17), *MUTYH* (*n* = 17), *PMS2* (*n* = 17), *SLX4* (*n* = 17), *POLE* (*n* = 16), *RAD50* (*n* = 15), *NF1* (*n* = 14), *PMS1* (*n* = 13), *MLH1* (*n* = 12), *MSH2* (*n* = 11), *PALB2* (*n* = 11), *BRIP1* (*n* = 10), and *FANCA* (*n* = 10); for all the other genes, less than 10 variants were reported according to our registry (Figure S1). The overall distribution of these genetic variants according to our initial classification within the ACMG/AMP categories was 16.64% as P (*n* = 100), 4.33% as LP (*n* = 26), 3% as P/LP (*n* = 18), and 76.04% as VUS (*n* = 457) (Figure 2a).

Using VarSome, the distribution changed at the expense of a lower proportion of VUS (288; 47.92%) with a higher proportion of P/LP (205; 34.11%) and B/LB variants (107; 17.80%) (Figure 2b); while with PathoMAN, the observed distribution was similar to our initial classification with a slightly lower proportion of VUS (445; 74.04%) and P/LP variants (117; 19.47%), and with a higher proportion of B/LB variants (26; 4.33%) (Figure 2c). It should be noted that PathoMAN classified four variants (0.67%) as Risk Factor/Founder. Also, the variant *APC*:c.‐30272A > G classified by us and by Invitae as VUS was not recognized either by VarSome or by PathoMAN, and the other eight variants were not recognized by PathoMAN.

**FIGURE 3:**
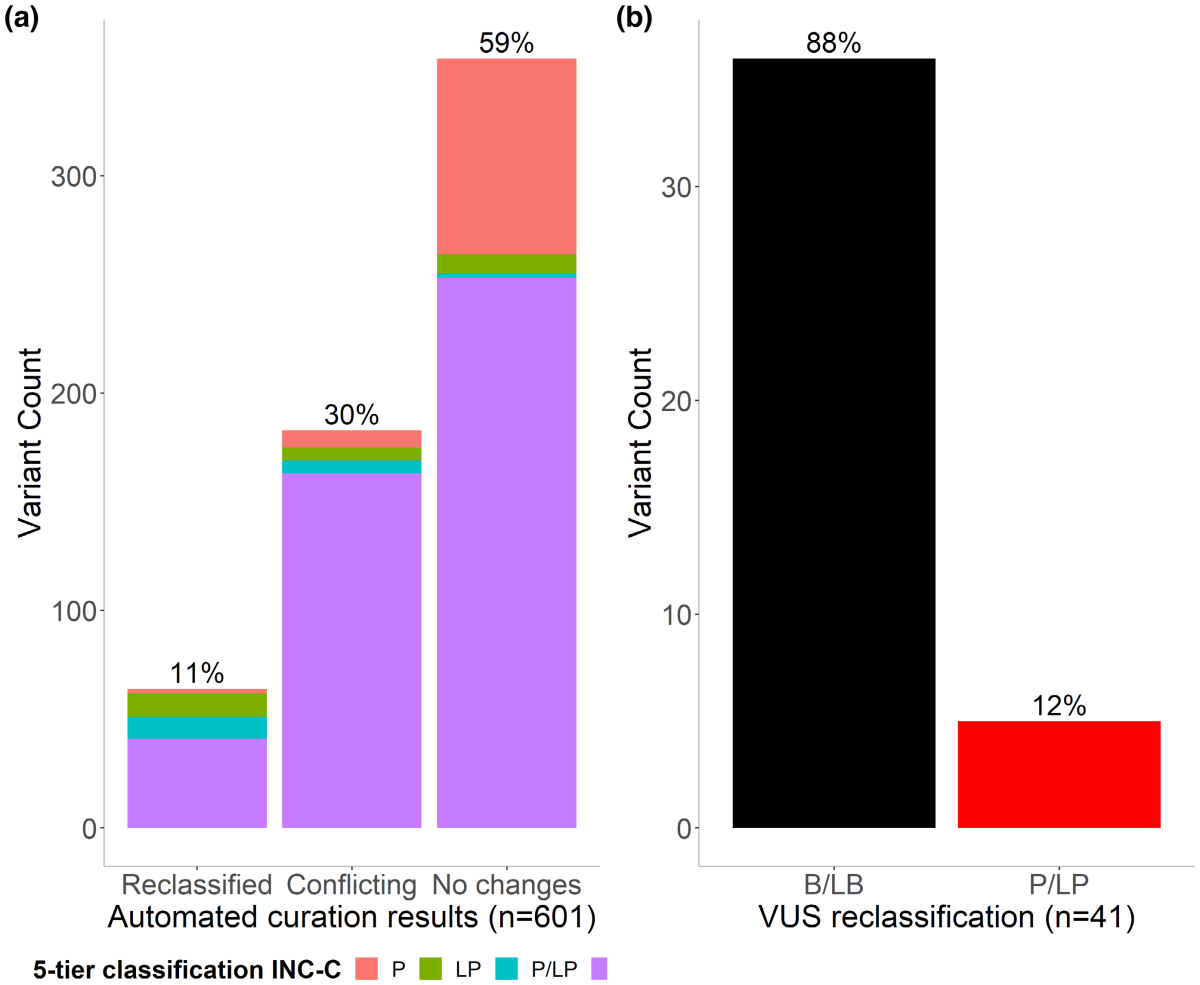
Conclusive end results of automated curation of 601 sequence variants. (a) Proportion of unique genetic variants reclassified; (b) Proportion of VUS downgraded and upgraded.

Although the conclusive end results of automated curation were mainly based on the reclassifications obtained by these two automated tools, a manual input was included for each variant which consisted in reviewing the criteria used by each tool and comparing them with evaluations made by experts in curated databases; for example, the variant NM_000546.6(*TP53*):c.869G > A;p.(Arg290His) was classified as a VUS by us and with VarSome and PathoMAN, but its final classification after automated curation was concluded as B due to the ClinGen expert panel report. In this sense, we obtained that 59% (354/601) of the variants did not change their interpretation, 11% (64/601) were reclassified, and 30% (183/601) presented a conflict of interpretation (Figures 1 and 3a). Most of the reclassified variants corresponded to VUS (41/64) that were downgraded to B/LB (88%; 36/41), while the minority were upgraded to P/LP (12%; 5/41) (Figure 3b).

**FIGURE 4:**
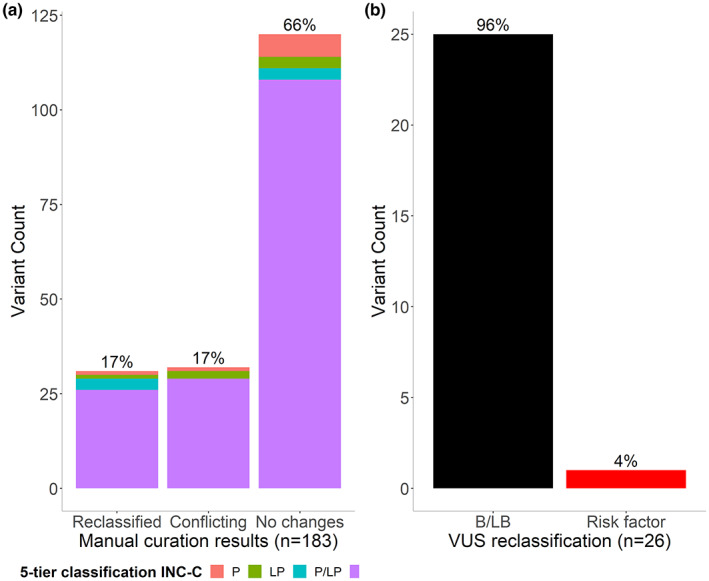
Conclusive end results of manual curation of 183 sequence variants with conflicting interpretation. (a) Proportion of unique genetic variants reclassified; (b) Proportion of VUS downgraded and upgraded.

Regarding the conclusive end results of manual curation of the variants with conflicting interpretation in the previous step, we obtained that 17% (31/183) of the variants were reclassified, 66% (120/183) did not change their interpretation compared to the initial classification in the Program's Registry, and 17% (32/183) remained with the conflicting interpretation status (Figure 4a and Table S2). Most of the reclassified variants after manual curation corresponded to variants initially classified by our team as VUS (26/31), and from these the majority were downgraded (96%; 25/26) to B/LB and only one variant (4%) was reclassified as “risk allele”, meaning an upgrade in its categorization (Figure 4b). This risk allele corresponds to the variant NM_001127511.3(*APC*):c.3920 T > A;p.(Ile1307Lys) (Zauber et al., 2014).

Overall, after applying the automated and manual curation workflow, 16% (95/601) of the variants were reclassified. From these, 71% (67/95) corresponded to VUS that were downgraded to less severe classifications (91%; 61/67) or upgraded to higher risk categories (9%; 6/67) (Table S3). Additionally, 29% (28/95) corresponded to P, LP, or P/LP variants reclassified; 7% (2/28) to less severe categories: the variant NM_004628.5(*XPC*):c.1001C > A;p.(Pro334His) changed from P to B and the variant NM_001005735.2(*CHEK2*):c.1556C > T;p.(Thr519Met) changed from LP to VUS. The other 93% (26/28) of these have negligible recategorization: 2 changed from P to LP; 11 changed from P/LP to P, 2 changed from P/LP to LP, and 11 changed from LP to P.

## 4 DISCUSSION

Germline variants can be reclassified over time as new evidence regarding their pathogenicity emerges, therefore, it is important to reassess periodically their clinical significance using up‐to‐date information since changes in its reclassification can have profound implications for patient care (Boonen et al., 2020; Djursby et al., 2020; Mighton et al., 2019; Slavin et al., 2019). This is especially important for VUS; thus, they consist of variants that cannot be classified as either P/LP or B/LB variants because the evidence is insufficient (Slavin et al., 2019). It is well‐recognized that the interpretation of variants is a challenging task since it must integrate information from different sources, determine frequency thresholds, and understand the clinical context (Preston et al., 2022; Sorrentino et al., 2021); for this reason, strategic planning is essential.

Even though the ACMG/AMP guidelines are the most widely used in the clinical setting for the classification of germline variants (Kim et al., 2019; Sorrentino et al., 2021), disagreements on variant pathogenicity classifications exist across laboratories (Amendola et al., 2020; Frone et al., 2021; Yang et al., 2017). Part of the discrepancy is due to the use of different technologies/tools and classification criteria, thus consensus‐building activities and data sharing are some of the recommendations to improve consistency in the variant interpretation process (Amendola et al., 2020). In accordance, within the last decade, there has been a great development in the field, with the design of different algorithms to comprehensively apply the ACMG/AMP evidence criteria (Preston et al., 2022; Sorrentino et al., 2021; Zhang et al., 2020) and with computational innovations related to the clinical interpretation of DNA sequence variants (Danos et al., 2019; Jimeno Yepes & Verspoor, 2014; Kopanos et al., 2019; Li & Wang, 2017; Pandey et al., 2012; Patel et al., 2017; Preston et al., 2022; Ravichandran et al., 2019; Tang & Thomas, 2016).

Here, we describe the reclassification rates of 601 unique sequence variants identified during 2 years through the Hereditary Cancer Program's Registry developed at the INC‐C, following the curation workflow defined by us. Although in this study our aim was not to give any conclusions regarding the best tool for automated or manual curation, we did evidence that the overall concordance of variant curation between VarSome and PathoMAN was 67.82%, and it slightly increased to 78.16% for variants in high penetrance genes such as *BRCA1, BRCA2, CDH1, PALB2, PTEN*, and *TP53* (data not shown). Part of the difference is evident in Figure 2, which shows the overall distribution of the genetic variants identified by us and their reclassification by each tool. Some explanations for this discrepancy could be the different bioinformatics approaches in interpreting the ACMG/AMP criteria by these tools and the insufficient clinical information for moderate‐ and low‐penetrance gene variants.

Other factors including the poor representation of Hispanic/Latino populations in genetic databases (Popejoy et al., 2018) along with its high genetic variability (Conomos et al., 2016) are important contributors to limitations in the variant curation process. It is well known that reference population databases are an essential tool for the interpretation of genomic variation. Thus, by increasing the representation of the Hispanic/Latino population, it is expected to achieve a better genetic characterization (i.e., allele frequency, per‐base expression levels, constraint scores, and variant co‐occurrence) that could be used to improve the clinical interpretation of candidate genetic variants in this ethnic group (Gudmundsson et al., 2022).

Population‐specific biobanks with repositories of genetic variants and health information are a valuable resource for supporting genotype–phenotype analyses and conclusion on clinical actionability. This was demonstrated by Whilhelm K., et al., when performing a secondary analysis of 161 genetic variants not reported in ClinVar complemented with longitudinal clinical data collected as part of a newborn screening program, which allowed the reclassification of 139 (86%) of these as P/LP (Wilhelm et al., 2022). It is noteworthy that an Institutional Biobank and a Program registry support our work with local genetic data and associated clinical information from cancer patients participating in the Hereditary Cancer Program at the INC‐C; in addition, these patients are being followed up by the Institutional Genetics Clinic. We strongly believe that manual curation and local genetic data can support future population‐specific variant curation exercises and genotype–phenotype analyses in our Colombian population.

The overall rate of variants reclassification in this study was 16% (95/601) similar to the 18% (268/1483) reported by Slavin T., et al, in a follow‐up period of 63 days to 20.2 years (median = 3.55 years) (Slavin et al., 2018). Among the 95 reclassified variants, 71% corresponded to VUS according to their initial classification recorded in the Program's Registry. In agreement with previous reports (Slavin et al., 2019), the majority of these were reclassified to a lower pathogenicity level (91%) and the minority to a higher pathogenicity level (9%). Reclassification of VUS as P/LP categories are rare but could be of high impact in the context of genomic cancer risk assessment and recommendations on cancer screening and management (Slavin et al., 2019). On the other hand, 29% (28/95) of the reclassified variants corresponded to P or LP or P/LP variants according to the Program's Registry, and 93% (*n* = 26) of these remained in the category of pathogenicity; specifically, 88% (*n* = 22) of the P/LP or LP variants reported (*n* = 25) were reclassified as P. This finding supports current clinical practice to treat LP variants similar to P variants and agrees with the ACMG/AMP guidelines that define the term “likely pathogenic” as having a probability greater than 90% of being “pathogenic” (Richards et al., 2015). Other studies show similar variant reclassification rates from LP to P (Harrison & Rehm, 2019; Slavin et al., 2018).

It should be noted that only 8/95 (8.4%) of the reclassified variants could change genetic counseling; 5/6 upgraded variants changed to P/LP, 1/6 as risk allele, and the remaining 2 were downgraded to VUS or B categories. The 5 VUS upgraded as P/LP, were identified in a heterozygous state in the genes *ERCC4* [(NM_005236.3:c.2395C) > T;p.(Arg799Trp)], *FANCD2* [(NM_001018115:c.990‐1G > A)], *FANCG* [(NM_004629.2:c.536 T) > G;p.(Leu179Ter)], *MUTYH* [(ENST00000528013.2:c.746G) > C;(p.Trp249Ser)], and *RAD50* [(NM_005732.4:c. 1970‐1G > T)]. Pathogenic variants in the *ERCC4* gene are associated with Fanconi anemia Group Q (MIM 615272), xeroderma pigmentosum group F (MIM 278760), xeroderma pigmentosum type F/Cockayne syndrome (MIM 278760), and progeroid syndrome XF (MIM 610965); *FANCD2* and *FANCG* are associated with Fanconi anemia Group D2 (MIM 227646) and Group C (MIM 614082), respectively; while *MUTYH* is associated with MUTYH‐associated polyposis (MIM 608456) and *RAD50* is associated with Nijmegen breakage syndrome‐like disorder (MIM 613078). Since all the above conditions have an autosomal recessive inheritance mechanism, neither of them was diagnosed with a hereditary cancer syndrome, meaning that these reclassifications did not alter medical management for the patients; nevertheless, the P or LP variants described previously may have reproductive implications, and options for planning pregnancy should be addressed during genetic counseling in these cases. Since P/LP variants in *RAD50* and *MUTYH* genes do not have sufficient evidence of its association with an increased risk of breast and/or ovarian cancer (Fan et al., 2018; Tung et al., 2016), no risk‐reducing measures should be offered to these carriers and risk assessment must be based on family history and other personal risk factors (NCCN Guidelines Version 1., 2023).

The VUS, reclassified as risk allele, corresponds to the variant NM_001127511.3(*APC*):c.3920 T > A;p.(Ile1307Lys) (Zauber et al., 2014) that has been reported as a common risk allele associated with familial colorectal cancer in the Ashkenazi Jewish population (Zauber et al., 2008, 2014) with a high frequency (6–7% among Ashkenazi Jews general population, 10–16% among Ashkenazi Jews with colon cancer, and 28% among those with family history of colorectal cancer) (Laken et al., 1997; Regev et al., 2005), but is not related with classic or attenuated familial adenomatous polyposis syndrome, FAP (MIM175100) (Regev et al., 2005). Moreover, an increased risk of colorectal cancer in individuals who are not of Ashkenazi Jewish ancestry has not been established (Boursi et al., 2013). According to the above, carriers of this risk allele in our cohort were not diagnosed with FAP, and screening recommendations for colorectal cancer should be based on family history.

Regarding the remaining 2/8 P/LP variants reclassified to a less severe category, the variant in the *XPC* gene [(NM_004628.5:c.1001C) > A;p.(Pro334His)] previously interpreted as P was downgraded as B; this variant was found in a heterozygous state, therefore, the patient was not diagnosed with xeroderma pigmentosum (MIM278720), and its reclassification did not modify the patient's diagnosis or management. The other variant in the *CHEK2* gene [NM_001005735.2:c.1556C > T;p.(Thr519Met)] previously interpreted as LP was downgraded to VUS; since for this gene only frameshift P/LP variants are considered as risk factors, the reclassification of this missense variant did not have profound effects on the medical care of the carriers; accordingly, screening recommendations for breast cancer are based on family history (Näslund‐Koch et al., 2016; NCCN Guidelines Version 1., 2023).

Overall, 5.32% (32/601) of the variants persisted with a conflicting interpretation status after automated and manual curation. The contradictory interpretation of genetic findings in clinical practice is frequent. Balmaña J., et al, carried out a registry of the results of hereditary cancer multigene panels from different laboratories, reporting contradictory interpretations in 26% of the interpreted variants, with greater frequency among the *CHEK2* and *ATM* genes, followed by *RAD51C*, *PALB2, BARD1, NBN*, and *BRIP1* (Balmaña et al., 2016). Similar to our results, many of the conflicting interpretations are of little clinical importance as discrepancies typically range between B/LB and VUS categories, and recommendations associated with some of these genes should be based on personal and family history (Balmaña et al., 2016). The recommendation of a follow‐up of at least every 2 years of potentially clinically relevant VUS should be taken into account since actionable reclassifications can occur during this period (Chiang et al., 2021).

Automation of variant curation enables fast processing and efficient delivery of reproducible results; however, this process requires manual interpretation by a human analyst in order to minimize errors inherent in the curation process (i.e., false‐positive and ‐negative results). For example, the variant NM_000546.6(*TP53*):c.869G > A;p.(Arg290His) previously annotated in our database as VUS and concordant with curation results by VarSome and PathoMAN was reclassified as B by ClinGen expert panel. Manual interpretation is usually tedious, repetitive, slow, and also prone to human error, which also explains the discordance between the classifications of germline variants between laboratories/groups. Therefore, a workflow with a unifying platform for the curation of germline sequence variants is necessary. In 2022, after our variant curation exercise was introduced “ClinGen Variant Curation Interface (VCI)”, a publicly available central platform for clinical variant classification according to the ACMG/AMP guidelines, which in turn enables collaboration and peer review across ClinGen Expert Panels supporting users in the process of making variant pathogenicity assertions (Preston et al., 2022). Future studies using VCI unifying platform are needed to evaluate if it could be a solution to avoid or minimize the existing variability in the interpretation of germline sequence variants.

Notwithstanding the limitations, our institutional experience and review of the literature show that: (i) genetic variants’ reclassification depends largely on the laboratories that offer the tests; (ii) most variants in the VUS category tend to be downgraded over time; and (iii) few reclassifications imply changes in actionability. We recommend that professionals actively involved in the performance and interpretation of molecular tests for clinical use work on the definition of standardized workflows. Delays or errors in the reclassification of variants can negatively affect shared decision‐making in relation to risk‐reducing measures, specific surgical management or targeted therapies, and follow‐up, as well as other factors related to genomic cancer risk assessment for the patient and relatives.

## 5 CONCLUSION

Here, we present the first genetic variant curation exercise conducted in the largest Institutional Hereditary Cancer Program in Colombia. In accordance with other studies, we obtained that most VUS were reclassified as B or LB (91%), while the rest were reclassified as P, LP, or risk factor. One influencing factor in the curation process defined in this study is the poor representation of genomic variability of Hispanic/Latino populations in international databases and research studies which makes this exercise challenging. Since false‐positive and ‐negative results can be obtained with automated tools, we recommend that manual curation should also be used as a complement. Finally, our results contribute to improving cancer risk assessment and management for a broad range of hereditary cancer syndromes in Hispanic/Latino populations.

## AUTHOR CONTRIBUTIONS

MCM and MCSS contributed to the study conception and design. ALR and MCSS performed the initial variant interpretation, and collected, registered, and revised the data included in the Institutional Hereditary Cancer Program registry. MCM performed the steps described for the variant curation exercise. The first draft of the manuscript was written by MCM and revised by MCSS. All the authors read and approved the final manuscript.

## FUNDING INFORMATION

MCSS (C19990300218—*Program for the Creation of a National Network of Hereditary Cancer in Colombia, INC‐C*) and MCSS (C19990300210—*Institutional Program for the Identification and Management of Families with Suspected Hereditary Cancer, INC‐C*).

## CONFLICT OF INTEREST

The authors have no conflicts of interest to declare.

## ETHICS STATEMENT

The Scientific Committee of the Instituto Nacional de Cancerología, Colombia (INC‐C), approved in 2017 the implementation of a Hereditary Cancer Program as a Quality Improvement Project to offer germline genetic testing as a standard‐of‐care service for cancer patients with suspicion of an underlying inherited condition.

## PATIENT CONSENT

Patients participating in this institutional Program accepted to be donors at the tissue Biobank from the INC‐C named “Banco Nacional de Tumores Terry Fox” and signed an informed consent for DNA biobanking and future research studies. The Program's registry is being implemented for epidemiological reports.

## WEB RESOURCES

Clinical Interpretations of Variants in Cancer, https://civic.genome.wustl.edu/. ClinVar, https://www.ncbi.nlm.nih.gov/clinvar/. ClinGen, https://erepo.clinicalgenome.org/evrepo/. Pathogenicity of Mutation Analyzer (PathoMAN), https://pathoman.mskcc.org. ClinGen Pathogenicity Calculator, https://calculator.clinicalgenome.org/site/cg‐calculator. Protein Bank, https://www.ncbi.nlm.nih.gov/protein/. GenBank, https://www.ncbi.nlm.nih.gov/genbank/. gnomAD, https://gnomad.broadinstitute.org/.

## Supporting information


**Figure S1** Genetic variants distribution by gene. Distribution of sequence variants (*n* = 601) submitted to our defined curation protocol per gene (*n* = 92)

**Table S1** Genes included in the multigene panel

**Table S2** Variants with conflicting interpretation

**Table S3** All VUS reclassified

## Data Availability

The published article includes part of the datasets generated or analyzed during this study as supplementary material. The complete dataset supporting the current study has not been deposited in a public repository but is available from the corresponding author upon reasonable request. This study did not generate a code for any bioinformatics' program.
