# Supplementary material for: Variant curation and interpretation in hereditary cancer genes: An institutional experience in Latin America
Source: Mol Genet Genomic Med. 2023 Mar 10;11(5):e2141. doi: 10.1002/mgg3.2141 (PMC10178801; doi:10.1002/mgg3.2141)
Supplement: Supplementary file 2 — Table S1 Genes included in the multigene panel [file MGG3-11-e2141-s003.docx]

**Table S1.** Genes included in the multigene panel

| *AIP* | *ALK* | *APC* | *ATM* | *BAP1* | *BARD1* | *BLM* | *BMPR1A* | *BRCA1* | *BRCA2* | *BRIP1* |
| --- | --- | --- | --- | --- | --- | --- | --- | --- | --- | --- |
| *BUB1B* | *CASR* | *CDC73* | *CDH1* | *CDK4* | *CDKN1B* | *CDKN1C* | *CDKN2A* | *CEBPA* | *CEP57* | *CHEK2* |
| *CYLD* | *DDB2* | *DICER1* | *DIS3L2* | *EGFR* | *EPCAM* | *ERCC2* | *ERCC3* | *ERCC4* | *ERCC5* | *EXT1* |
| *EXT2* | *EZH2* | *FANCA* | *FANCB* | *FANCC* | *FANCD2* | *FANCE* | *FANCF* | *FANCG* | *FANCI* | *FANCL* |
| *FANCM* | *FH* | *FLCN* | *GATA2* | *GNAS* | *GPC3* | *HNF1A* | *HRAS* | *KIT* | *MAX* | *MEN1* |
| *MET* | *MLH1* | *MRE11A* | *MSH2* | *MSH6* | *MUTYH* | *NBN* | *NF1* | *NF2* | *NSD1* | *PALB2* |
| *PDE4D* | *PHOX2B* | *PMS1* | *PMS2* | *POLD1* | *POLE* | *PPM1D* | *PRF1* | *PRKAR1A* | *PTCH1* | *PTEN* |
| *RAD50* | *RAD51C* | *RAD51D* | *RB1* | *RECQL4* | *RET* | *RHBDF2* | *RUNX1* | *SBDS* | *SDHA* | *SDHAF2* |
| *SDHB* | *SDHC* | *SDHD* | *SLX4* | *SMAD4* | *SMARCB1* | *STK11* | *SUFU* | *TMEM127* | *TP53* | *TSC1* |
| *TSC2* | *VHL* | *WRN* | *WT1* | *XPA* | *XPC* |  |  |  |  |  |
